# Type-2-Diabetes Alters CSF but Not Plasma Metabolomic and AD Risk Profiles in Vervet Monkeys

**DOI:** 10.3389/fnins.2019.00843

**Published:** 2019-08-28

**Authors:** Kylie Kavanagh, Stephen M. Day, Morgan C. Pait, William R. Mortiz, Christopher B. Newgard, Olga Ilkayeva, Donald A. Mcclain, Shannon L. Macauley

**Affiliations:** ^1^Department of Pathology, Wake Forest School of Medicine, Winston-Salem, NC, United States; ^2^College of Health and Medicine, University of Tasmania, Hobart, TAS, Australia; ^3^Section of Gerontology and Geriatric Medicine, Department of Internal Medicine, Wake Forest School of Medicine, Winston-Salem, NC, United States; ^4^Department of Neurology, Washington University School of Medicine, St. Louis, MO, United States; ^5^Sarah W. Stedman Nutrition and Metabolism Center, Duke Molecular Physiology Institute, Duke University Medical Center, Durham, NC, United States; ^6^Section of Endocrinology and Metabolism, Wake Forest School of Medicine, Winston-Salem, NC, United States

**Keywords:** metabolomics, type 2 diabetes, Alzheimer’s disease, amyloid-beta, CSF, amino acids, acylcarnitine, hyperglycemia

## Abstract

Epidemiological studies suggest that individuals with type 2 diabetes (T2D) have a twofold to fourfold increased risk for developing Alzheimer’s disease (AD), however, the exact mechanisms linking the two diseases are unknown. In both conditions, the majority of pathophysiological changes, including glucose and insulin dysregulation, insulin resistance, and AD-related changes in Aβ and tau, occur decades before the onset of clinical symptoms and diagnosis. In this study, we investigated the relationship between metabolic biomarkers associated with T2D and amyloid pathology including Aβ levels, from cerebrospinal fluid (CSF) and fasting plasma of healthy, pre-diabetic (PreD), and T2D vervet monkeys (*Chlorocebus aethiops sabaeus*). Consistent with the human disease, T2D monkeys have increased plasma and CSF glucose levels as they transition from normoglycemia to PreD and diabetic states. Although plasma levels of acylcarnitines and amino acids remained largely unchanged, peripheral hyperglycemia correlated with decreased CSF acylcarnitines and CSF amino acids, including branched chain amino acid (BCAA) concentrations, suggesting profound changes in cerebral metabolism coincident with systemic glucose dysregulation. Moreover, CSF Aβ_*40*_ and CSF Aβ_*42*_ levels decreased in T2D monkeys, a phenomenon observed in the human course of AD which coincides with increased amyloid deposition within the brain. In agreement with previous studies in mice, CSF Aβ_*40*_ and CSF Aβ_*42*_ were highly correlated with CSF glucose levels, suggesting that glucose levels in the brain are associated with changes in Aβ metabolism. Interestingly, CSF Aβ_*40*_ and CSF Aβ_*42*_ levels were also highly correlated with plasma but not CSF lactate levels, suggesting that plasma lactate might serve as a potential biomarker of disease progression in AD. Moreover, CSF glucose and plasma lactate levels were correlated with CSF amino acid and acylcarnitine levels, demonstrating alterations in cerebral metabolism occurring with the onset of T2D. Together, these data suggest that peripheral metabolic changes associated with the development of T2D produce alterations in brain metabolism that lead to early changes in the amyloid cascade, similar to those observed in pre-symptomatic AD.

## Introduction

Rates of type 2 diabetes (T2D) and Alzheimer’s disease (AD) are reaching epidemic proportions and are expected to continue to rise over the next several decades (Holtzman et al., 2011). T2D is a metabolic disorder characterized by elevated fasting plasma glucose levels, increased insulin levels, insulin resistance, and beta cell dysfunction with the majority of changes occurring 5–10 years before clinical diagnosis (American Diabetes Association, 2010). Similarly, pathological hallmarks of AD, including the extracellular aggregation of amyloid β (Aβ) into amyloid plaques and the intracellular accumulation of the tau protein into neurofibrillary tangles (NFTs), begin decades before cognitive decline and clinical diagnosis (Hardy and Selkoe, 2002; Bateman et al., 2012; Musiek and Holtzman, 2015). While both are considered diseases of aging and mechanisms linking the two conditions remain elusive, epidemiological and cross-sectional studies suggest that individuals with T2D have a twofold to fourfold increased risk for developing AD and dementia and show increased AD pathology (Ott et al., 1999; Crane et al., 2013; Huang et al., 2014). Preclinical studies in mouse models of cerebral amyloidosis suggest that systemic hyperglycemia increases Aβ levels within the hippocampal interstitial fluid (ISF) by 25%; an effect that is amplified when plaques are already present in the brain during the hyperglycemia challenge (Macauley et al., 2015; Stanley et al., 2016). Moreover, mouse plasma glucose, ISF glucose, and ISF Aβ are highly correlated, and elevated glucose levels drive Aβ production in the hippocampus in an activity dependent manner (Macauley et al., 2015). Conversely, systemic hyperinsulinemia at post-prandial or supra-physiological levels, only modestly increase ISF Aβ levels. This suggests that changes in glucose, rather than insulin, correlate more closely with brain Aβ levels (Stanley et al., 2016). Although these studies suggest a mechanistic link between T2D and AD, rodent models of AD do not fully recapitulate the human course of disease, and it is important to translate these observations to primates. Similar to humans, many non-human primate species develop T2D and amyloid pathology with age (Wagner et al., 2006; Latimer et al., 2019), and thus represent an important translational tool for examining the metabolic relationship between the two conditions.

Branched chain amino acids (BCAAs), including leucine, isoleucine, and valine, are essential amino acids necessary for protein synthesis, but when found in excess, impact energy homeostasis (Shimomura and Kitaura, 2018; Hudd et al., 2019; White and Newgard, 2019). Recent work demonstrated that elevated dietary BCAA intake is associated with obesity and insulin resistance in both humans and rodents (Newgard et al., 2009; Solon-Biet et al., 2019), and plasma BCAA levels are highly predictive of T2D development in normoglycemic individuals (Wang et al., 2011). Elevated levels of circulating BCAAs are associated with suppressed mitochondrial β-oxidation, reduced glucose tolerance, increased insulin resistance, and increased *de novo* lipogenesis, making BCAAs a potential biomarker of metabolic disease (Newgard et al., 2009; Weiss and Lustig, 2014). BCAAs are also integral to healthy brain function, due to their roles in neurotransmitter biosynthesis, protein synthesis, and energy production. Alterations in BCAA levels in plasma and CSF have been implicated in AD pathology, with conflicting evidence on whether they are helpful or harmful to disease progression (Griffin and Bradshaw, 2017). Nevertheless, alterations in energy homeostasis and BCAA catabolism represent one potential link between T2D and AD.

Acylcarnitines are byproducts of mitochondrial fatty acid, amino acid and glucose catabolism that serve as useful biomarkers of metabolic changes (Jones et al., 2010). Changes in the plasma acylcarnitine profile have been observed in obesity, T2D, and insulin resistance, representing alterations in several metabolic pathways (Jones et al., 2010; Schooneman et al., 2013). Moreover, acylcarnitines are key energy substrates in the brain, especially during fasting conditions when glucose levels are low (Jones et al., 2010). In AD patients, plasma levels of acylcarnitines are decreased, suggesting perturbations in energy metabolism that may be central to AD pathogenesis (Cristofano et al., 2016).

Here, we applied comprehensive metabolic profiling tools to healthy control (Ctrl), pre-diabetic (PreD), and diabetic (T2D) monkeys to explore the relationship between T2D and amyloid pathology. We utilized a cohort of aging vervet monkeys (*Chlorocebus aethiops sabaeus)*, which develop neuropathological changes consistent with human AD pathology including increased amyloid plaque burden, elevated cortical tau levels and paired helical filament tau immunoreactivity, Aβ-related vascular impairment, reduced cerebral metabolism, regional atrophy, decreased CSF Aβ_*42*_ and increased CSF tau levels (Kalinin et al., 2013; Chen et al., 2018; Latimer et al., 2019), to ensure translational relevance of our findings. We analyzed plasma and CSF amino acids and acylcarnitine concentrations and explored how these changes related to CSF Aβ_*40*_ and Aβ_*42*_ levels, which are established biomarkers of disease in AD.

## Materials and Methods

### Animals

The monkeys used in this study were sourced from a multigenerational pedigreed colony of vervet monkeys (*Chlorocebus aethiops sabaeus*; age = 16.5–23.5 years old). Veterinary and research staff categorized the monkeys as either healthy (Ctrl; *n* = 4), pre-diabetic (PreD; *n* = 4), or type-2 diabetic (T2D; *n* = 5) according to repeated fasting glucose measurements and American Diabetes Association criteria (American Diabetes Association, 2010) and were selected to be matched by age, bodyweight, and adiposity as measured by waist circumference. PreD and T2D categorization was only made after ≥2 consecutive fasting glucose values were ≥100 mg/dL or ≥126 mg/dL, respectively. T2D monkeys were maintained with insulin therapy, and all T2D monkeys in study had been diagnosed and treated for a minimum of 2 years. Monkeys were fed a commercial laboratory primate chow diet (Laboratory Diet 5038; LabDiet., St. Louis, MO, United States), with daily supplemental fresh fruits and vegetables. This standard laboratory diet is comprised of 13% calories from fat; 69% calories from carbohydrates; and 18% of calories from protein. All samples were collected after 16 h fasting and withdrawal from all exogenous insulin. Cerebrospinal fluid (CSF) was collected via puncture of the atlanto-occipital space, and plasma samples were collected from the femoral vein.

All animal procedures were performed on a protocol approved by the Wake Forest University Institutional Animal Care and Use Committee according to recommendations in the Guide for Care and Use of Laboratory Animals (Institute for Laboratory Animal Research) and in compliance with the USDA animal Welfare Act and Animal Welfare Regulations (Animal Welfare Act as Amended; Animal Welfare Regulations).

### AD Biomarkers

Aβ_40_ and Aβ_42_ levels from CSF samples were assayed using sandwich ELISAs as previously described (Bero et al., 2011; Roh et al., 2012). Briefly, Aβ_40_ and Aβ_42_ were quantified using monoclonal capture antibodies (a generous gift from David Holtzman) targeted against amino acids 45–50 (HJ2) or 37–42 (HJ7.4), respectively. For detection, both Aβ_*40*_ and Aβ_*42*_ used a biotinylated monoclonal antibody against the central domain (HJ5.1B), followed by incubation with streptavidin-poly-HRP-40. Assays were developed using Super Slow TMB (Sigma) and the plates read on a Bio-Tek Synergy 2 plate reader at 650 nm.

### Metabolomics, Lipids, Glucose, and Lactate Measures

Glucose and lactate measurements within the plasma and CSF were quantified using a YSI 2900 analyzer as previously described (Macauley et al., 2015). A detailed description of blood and CSF sample preparation and coefficients of variation for these assays has been published (Haqq et al., 2005; Solon-Biet et al., 2019). Insulin was measured by ELISA (Mercodia, Uppsala, Sweden) in plasma and CSF samples. Total cholesterol, high density lipoprotein (HDL) and low-density lipoprotein (LDL) cholesterol, and triglycerides were measured with kits from Roche Diagnostics (Indianapolis, IN, United States) and free fatty acids (total) and ketones (total and 3-hydroxybutyrate) with kits from Wako (Richmond, VA, United States). ApoB associated cholesterol was calculated as the total cholesterol minus HDLC. Plasma and CSF acylcarnitines and amino acids were analyzed by MS/MS as described previously (Millington et al., 1990; Chace et al., 1995; An et al., 2004; Wu et al., 2004; Ferrara et al., 2008).

### Data Analysis

Data were analyzed using one-way ANOVA and correlations were determined by Pearson’s correlation coefficient, *r*. To determine the relative relationship between CSF Glucose, CSF Aβ_42_, and plasma lactate and CSF analytes, we transformed each data point to represent its value relative to the control group mean [% control mean value = 100^∗^(*x*/control mean), where *x* = any given data point]. Data are represented as means ± SEM. Tukey’s *post hoc* tests were used when appropriate.

## Results

### Metabolic Profile of Normoglycemic, Pre-diabetic, and T2D Monkeys

Monkeys were older, ranging from 16 to 23 years (Table 1), which represents the last 30% of lifespan for this species and is a typical age range for the onset of metabolic diseases and neuropathological changes associated with AD (Kalinin et al., 2013; Chen et al., 2018; Latimer et al., 2019). There were no differences in body weight or waist circumference between groups (Table 1). PreD and T2D monkeys had elevated fasting blood glucose levels compared to normoglycemic controls [Table 1; *p* < 0.0001, *F*(2,9) = 39.17], but there were no differences in fasting insulin levels (Table 1). While HOMA scores were elevated in PreD and T2D monkeys, the differences were not significant (Table 1). Additionally, lipid measures illustrated higher triglycerides in T2D monkeys compared to PreD or Ctrl. Together, elevated fasting blood glucose was the most notable finding delineating the Ctrl, PreD, and T2D cohorts.

**TABLE 1:** Demographic and metabolic characteristics of monkeys included in study.

	**Ctrl Mean (SEM)**	**PreD Mean (SEM)**	**T2D Mean (SEM)**	**One-Way ANOVA**
Age (years)	20.25 (±1.109)	18.75 (±0.6292)	18.00 (±1.304)	*p* = 0.3800
Body weight (kg)	5.553 (±0.5525)	6.618 (±0.9210)	5.414 (±0.4937)	*p* = 0.4100
Waist circumference (cm)	36.98 (±1.719)	41.94 (±3.861)	38.08 (±3.441)	*p* = 0.5283
Fasting glucose (mg/dL)	67.84 (±5.097)	113.80 (±4.308)	161.10 (±11.05)	*p* < 0.0001
Fasting insulin (μlU/mL)	20.83 (±4.806)	37.43 (±8.854)	26.63 (±8.191)	*p* = 0.3284
HOMA Score (AU)	3.613 (±1.048)	10.61 (±2.721)	10.64 (±3.525)	*p* = 0.1512
TPC (mg/dL)	171.3 (±10.06)	198.9 (±33.75)	164.3 (±9.067)	*p* = 0.4945
Triglyceride (mg/dL)	74.33 (±2.541)	59.75 (±7.417)	103.80 (±16.19)	*p* = 0.0407
HDLC (mg/dL)	60.59 (±4.389)	65.50 (±5.939)	59.83 (±3.657)	*p* = 0.6702
ApoB-associated cholesterol (mg/dL)	110.70 (±6.232)	95.22 (±8.152)	104.40 (±6.809)	*p* = 0.3633
TPC/HDLC	2.845 (±0.093)	2.360 (±0.130)	2.785 (±0.148)	*p* = 0.0646

*Ctrl = Control; PreD = Pre-Diabetic; T2D = Type-2 Diabetic; HOMA = Homeostatic model assessment. TPC = Total plasma cholesterol; HDLC = High-density lipoprotein cholesterol.*

### Increased CSF Glucose Levels Correlated With Decreased CSF Aβ_40_ and Aβ_42_ Concentrations in T2D Monkeys

**FIGURE 1:**
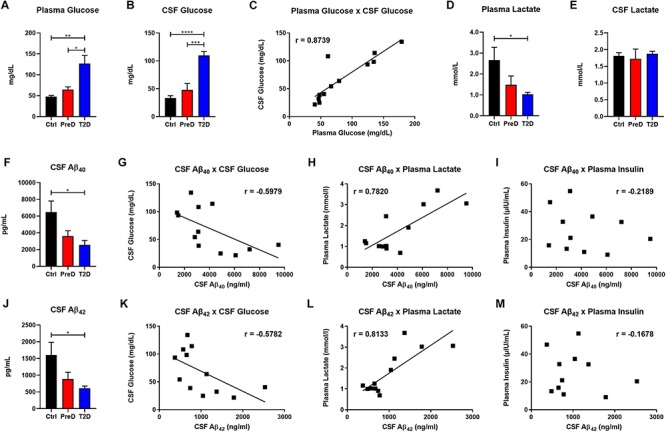
Type 2 diabetes (T2D) monkeys have significantly decreased cerebrospinal fluid (CSF) Aβ_40_ and CSF Aβ_42_ concentrations, which is correlated with CSF glucose and plasma lactate concentrations. **(A)** plasma glucose and **(B)** CSF glucose concentrations were significantly elevated in T2D monkeys. **(C)** Plasma and CSF glucose concentrations were significantly correlated. **(D)** T2D monkeys had significantly decreased plasma lactate, but not **(E)** CSF lactate. **(F)** T2D monkeys had significantly decreased Aβ_40_, which was correlated with **(G)** CSF glucose and **(H)** plasma lactate, but not **(I)** plasma insulin concentrations. **(J)** Similarly, T2D monkeys had significantly decreased Aβ_42_ concentrations, which was correlated with **(K)** CSF glucose and **(L)** plasma lactate, but not **(M)** plasma insulin concentrations.^∗^*p* < 0.05; ^∗∗^*p* < 0.01; ^∗∗∗^*p* < 0.001; and ^∗∗∗∗^*p* < 0.0001; one-way ANOVA with Tukey’s *post hoc* test. Values represent mean ± SEM; solid lines represent statistically significant correlations (*p* < 0.05).

T2D monkeys have elevated plasma glucose [Figure 1A; *p* = 0.0036, *F*(2,10) = 10.39] and CSF glucose concentrations [Figure 1B; *p* < 0.0001, *F*(2,9) = 32.31] compared to normoglycemic controls, while PreD had intermediate values. Data analysis revealed that plasma and CSF glucose levels had a strong positive correlation (Figure 1C; *p* < 0.0002, *r* = 0.8739, *R*^2^ = 0.7637), which is consistent with observations from preclinical rodent models (Macauley et al., 2015). We also observed that plasma lactate levels were lower in T2D monkeys [Figure 1D; *p* = 0.0395, *F*(2,10) = 4.544], however, no differences in CSF lactate concentrations were observed (Figure 1E). We previously demonstrated that hyperglycemic APP/PS1 mice, a model of cerebral amyloidosis, have elevated Aβ within the brain’s ISF (Macauley et al., 2015; Stanley et al., 2016), and non-human primates with T2D have increased Aβ deposition in several brain regions (Okabayashi et al., 2015). T2D monkeys have decreased CSF Aβ_40_ [Figure 1F; *p* = 0.0280, *F*(2,9) = 5.030], and CSF Aβ_42_ concentrations [Figure 1J; *p* = 0.0342, *F*(2,9) = 5.463]. Interestingly, both CSF Aβ_40_ and CSF Aβ_42_ were highly correlated with CSF glucose (Figure 1G; *p* = 0.0400, *r* = −0.5979, *R*^2^ = 0.3575 and Figure 1K; *p* = 0.0489, *r* = −0.5782, *R*^2^ = 0.3343, respectively) and plasma lactate (Figure 1H; *p* = 0.0027, *r* = 0.7820, *R*^2^ = 0.6115 and Figure 1L *p* = 0.0013, *r* = 0.8133, *R*^2^ = 0.6614; respectively) but not with plasma insulin (Figures 1I,M). CSF insulin levels were undetectable. Given that decreased CSF Aβ is indicative of increased plaque formation within the brain (Tapiola et al., 2009), these data indicate that T2D monkeys display biomarkers of early amyloid deposition and pre-symptomatic AD (Kalinin et al., 2013; Chen et al., 2018; Latimer et al., 2019) triggered by a state of energy dysregulation, which is consistent with previous work in rodent models (Macauley et al., 2015; Stanley et al., 2016).

### T2D and PreD Monkey Show Decreased Amino Acid and Acylcarnitines Levels in the CSF but Not the Plasma

**FIGURE 2:**
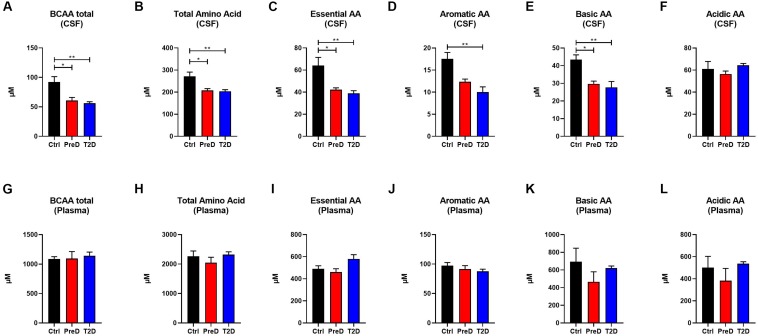
Amino acid(AA) concentrations are decreased in the cerebrospinal fluid (CSF) but not plasma of type 2 diabetes (T2D) monkeys. **(A)** T2D monkeys had significantly decreased branch chain amino acids (BCAA), **(B)** total amino acid concentrations, **(C)** essential AAs, **(D)** aromatic AAs, and **(E)** basic AAs, but not **(F)** acidic AAs. **(G–L)** Conversely, plasma amino acid concentrations were not different between groups. ^∗^*p* < 0.05; ^∗∗^*p* < 0.01; ^∗∗∗^*p* < 0.001; and ^∗∗∗∗^*p* < 0.0001; one-way ANOVA with Tukey’s *post hoc* test. Values represent mean ± SEM.

Because plasma amino acid and acylcarnitine levels are linked to metabolic dysfunction in T2D (Schooneman et al., 2013; Shin et al., 2014), the levels of amino acids (AA) in both the plasma and CSF were quantified to further explore the energy imbalance associated with T2D. In examining the AA concentrations by their functional groups, PreD and T2D monkeys had lower concentrations of amino acids in the CSF. Amino acids were further stratified into branched-chain AA [BCAAs, Figure 2A; *p* = 0.0038, *F*(2,9) = 11.04], total AA [Figure 2B; *p* = 0.0081, *F*(2,9) = 8.638], essential AA [Figure 2C; *p* = 0.0065, *F*(2,9) = 9.280], aromatic AA [Figure 2D; *p* = 0.0046, *F*(2,9) = 10.36], and basic AA [Figure 2E; *p* = 0.0087, *F*(2,9) = 8.421] (Supplementary Table S1). In the CSF, all AA groups were lower in T2D, with the exception of acidic AA where no changes were detected (Figure 2F). No differences in plasma AA concentrations were detected in any of the AA categories (Figures 2G–L). Interestingly, when individual AAs were measured in plasma (Supplementary Table S3 and Supplementary Figure S2), there was a trend toward an increase in levels of the BCAAs valine and leucine/isoleucine (Supplementary Figure S2F; *p* = 0.0977 and Supplementary Figure S2M; *p* = 0.1675, respectively), suggesting peripheral metabolic perturbations were present, although the differences in CSF amino acids (Supplementary Figures S1A–O and Supplementary Table S4), valine and leucine/isoleucine in particular, were more striking (Supplementary Figure S1F; *p* = 0.0219 and Supplementary Figure S1M; *p* = 0.0524).

**FIGURE 3:**
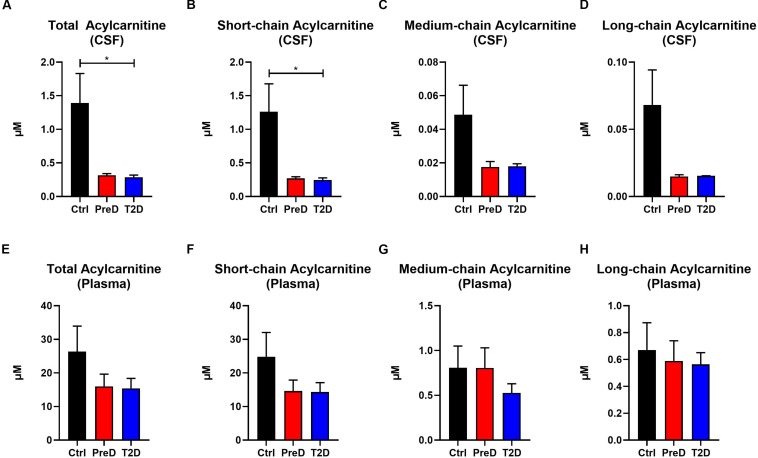
Acylcarnitine concentrations are decreased in the cerebrospinal fluid (CSF) but not plasma of type 2 diabetes (T2D) monkeys. **(A,B)** Total and short-chain acylcarnitine concentrations were significantly decreased in the plasma of T2D and IR monkeys, **(C,D)**, however, medium- and long-chain acylcarnitines were not significantly different. **(E–H)** There were no differences in plasma acylcarnitine concentrations between groups.^∗^*p* < 0.05; one-way ANOVA with Tukey’s *post hoc* test. Values represent mean ± SEM.

Acylcarnitines are derived from the mitochondrial oxidation of fatty acids, carbohydrates, and amino acids (Schooneman et al., 2013). Several studies have shown that T2D patients have elevated plasma acylcarnitine concentrations compared to healthy controls (Muoio, 2014). Here, T2D monkeys had lower total acylcarnitine concentrations in the CSF [Figure 3A; *p* = 0.0208, *F*(2,9) = 6.139], but no differences in plasma total acylcarnitine levels (Figure 3C), a pattern consistent with the AA data (Supplementary Table S2). In the CSF, T2D monkeys also had lower short-chain acylcarnitine concentrations [Figure 3B; *p* = 0.0245, *F*(2,9) = 5.764]. However, due to variability in the control monkeys, medium- and long-chain acylcarnitine concentrations were consistently lower in T2D monkeys, but the difference did not reach significance (Muoio, 2014; Figures 3C,D). Again, plasma concentrations remained comparable between groups (Figures 3F–H). Together, this data suggests that fuel metabolism is altered in the brains of T2D monkeys compared to normoglycemic controls.

### Metabolic Dysregulation in the Brain Is Associated With Changes in CSF Glucose, Plasma Glucose, CSF Aβ_42_, and Plasma Lactate

**FIGURE 4:**
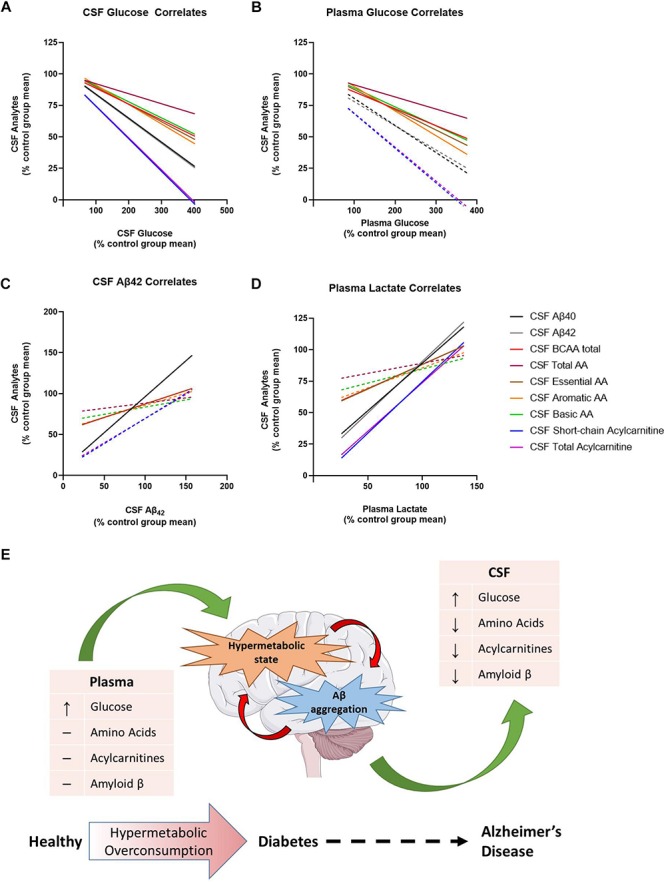
Metabolic dysregulation is associated with changes in plasma lactate, cerebrospinal fluid (CSF) glucose, and CSF Aβ_40_ and Aβ_42_. Here, each data point has been converted into a value that represents the % of the control group’s mean [value = 100^∗^(*x*/control group mean)]. **(A)** Normalized CSF glucose values were negatively correlated with several normalized CSF analytes. **(B)** Normalized plasma glucose were negatively correlated with normalized CSF amino acids, but not normalized CSF Aβ_40__,_ Aβ_*42*_, or acylcarnitines. **(C)** Normalized CSF Aβ_42_ was correlated with normalized CSF analytes. **(D)** Normalized plasma lactate values were positively correlated with several normalized CSF analytes. **(E)** Thus, the model we propose here is that type 2 diabetes (T2D) moves the brain into a state of hypermetabolic overconsumption wherein the brain consumes increased energy, leading to lower concentrations of Aβ_42_ in the CSF, which is indicative of increased Aβ aggregation. Lines represent non-linear regression best fit curve; solid lines indicate statistical significance.

Lastly, we investigated the relationship between differences in CSF amino acids and CSF acylcarnitines as a function of CSF glucose, CSF Aβ_42_, and plasma lactate concentrations (Figure 4) to further elucidate the interaction between early metabolic changes in T2D with early biomarker alterations in AD. There was an overall negative relationship between CSF glucose and Aβ_40_, Aβ_42_, total AA, essential AA, BCAA, aromatic AA, basic AA, short-chain acylcarnitine, and total acylcarnitine (Figure 4A). Thus, as CSF glucose increases as observed in PreD and T2D, concentrations of amino acids, acylcarnitines, and Aβ all decrease in the CSF. Plasma glucose was correlated with CSF BCAA, total AA, essential AA, aromatic AA, and basic AA, but not CSF Aβ_40_, CSF Aβ_42_, or CSF acylcarnitines (Figure 4B and Supplementary Table S6). Next, CSF Aβ_42_ was correlated with CSF Aβ_40_ and CSF BCAA (Figure 4C), reinforcing the relationship between BCAAs and amyloid pathology. Lastly, plasma lactate concentrations correlated with CSF Aβ_40_ and Aβ_42_, CSF essential AA and BCAA, and CSF short-chain and total acylcarnitine concentrations (Figure 4D), demonstrating plasma lactate might be a potential biomarker for early changes in T2D and pre-symptomatic AD. Taken together, alterations in cerebral metabolism co-vary with changes in plasma glucose, plasma lactate, and CSF Aβ_*42*_ which highlight the importance of metabolic changes in the pathogenesis of T2D and AD.

## Discussion

In this study, elevated fasting blood glucose levels associated with the onset of T2D elicit changes in brain metabolism and correlate with changes in the amyloid cascade, an early indicator of presymptomatic AD (Crane et al., 2013; Macauley et al., 2015). Aged monkeys (>19 years) can develop pathology consistent with human AD including increased amyloid plaque burden, elevated cortical tau levels and paired helical filament tau immunoreactivity, Aβ-related vascular damage, reduced cerebral metabolism, regional atrophy, and alterations in CSF biomarkers including both Aβ and tau (Kalinin et al., 2013; Chen et al., 2018; Latimer et al., 2019). Moreover, reductions in CSF Aβ levels correlate with increased amyloid plaque burden and insoluble Aβ levels in the cortex, similar to findings from human studies. In this study, T2D monkeys had lower CSF Aβ_*40*_ and Aβ_*42*_ levels, which is indicative of increased amyloid deposition within the brain (Tapiola et al., 2009). In agreement with previous rodent studies (Macauley et al., 2015; Stanley et al., 2016), CSF Aβ_40_ and Aβ_42_ were highly correlated with CSF glucose levels, which suggests that increased glucose may be driving Aβ production and aggregation in these animals. Interestingly, CSF Aβ_40_ and Aβ_42_ levels also highly correlated with plasma lactate levels, which is consistent with published studies that show decreased plasma lactate correlates with AD severity (Lu et al., 2015; Verri et al., 2018). Moreover, T2D vervet monkeys had lower CSF acylcarnitine and CSF amino acids, while plasma levels were largely unchanged, suggesting either early changes in cerebral metabolism with the onset of T2D or changes in the transport of certain metabolic fuels to the brain. Reduced CSF Aβ_40_ and Aβ_42_ levels in T2D monkeys correlated with higher plasma and CSF glucose concentrations, suggesting increased amyloid plaques are related to glucose dysregulation. Lastly, we showed that CSF amino acids and acylcarnitines were negatively correlated with CSF glucose and positively correlated with CSF Aβ_40_, CSF Aβ_42_, and plasma lactate. Together, these data suggest that peripheral metabolic changes associated with diabetogenesis co-occur with alterations in brain metabolism. Moreover, these metabolic changes are associated with activation of the amyloid cascade typically observed in humans with pre-symptomatic AD. Future postmortem studies in T2D monkeys should examine tau hyperphosphorylation, NFTs, and amyloid plaques in addition to changes in CSF Aβ levels to further strengthen the connection between T2D and AD pathology.

Our data further supports existing evidence that chronic hyperglycemia and metabolic dysfunction are a pathological link between T2D and AD. In humans, hyperglycemia increases dementia risk in both patients with and without diabetes, causes rapid progression from mild cognitive impairment (MCI) to symptomatic AD, and increases the rate of amyloid accumulation in the brain (Crane et al., 2013; Morris et al., 2014). Moreover, hyperglycemia and increased HbA1c levels correlate with memory impairment, decreased functional connectivity, and increased neuronal loss, independent of T2D or AD diagnosis (Zheng et al., 2018). Data from T2D monkeys illustrates the same phenomenon; elevated blood glucose levels increase CSF glucose which correlates with changes in CSF Aβ levels, presumably due to the sequestration of Aβ into amyloid plaques in the brain (Bateman et al., 2006). Our findings in the T2D monkeys also uncovered an interesting relationship between glucose, lactate, and Aβ which supports previous findings from rodent studies. Preclinical studies in mouse models of cerebral amyloidosis demonstrate that synaptic release of Aβ occurs in an activity dependent manner, where high levels of synaptic activity increase Aβ secretion (Cirrito et al., 2003; Cirrito et al., 2005; Bateman et al., 2006; Cirrito et al., 2008). Increased synaptic activity not only drives ISF Aβ release but also the release of lactate into the extracellular space (Bero et al., 2011). According to the astrocyte neuron lactate shuttle, lactate is a preferred energy source for neurons to sustain excitatory neurotransmission and levels of ISF lactate correlate with neuronal activity. Our previous work demonstrated that hyperglycemia not only increases ISF glucose and ISF Aβ but also ISF lactate, illustrating that increased metabolic activity is linked with increased synaptic activity and Aβ release. Since a direct measure of lactate production in the brain of T2D monkeys was unattainable in this study, we explored how plasma and CSF levels changed with peripheral hyperglycemia. Interestingly, plasma lactate, but not CSF lactate, correlated with changes in CSF glucose and Aβ. In accordance with our previous work, we hypothesize that increased glucose metabolism is increasing neuronal activity within the brain and driving both the production of Aβ and the consumption of pyruvate and lactate as fuel. Although the changes in plasma lactate levels could be due to alterations in peripheral metabolism in the T2D monkeys, we propose a different mechanism where increased lactate consumption in the brain signifies a hyperactive and hypermetabolic brain state present in both T2D and AD (Figure 4E). Since the concentration gradient for lactate favors transport from brain to blood (Raichle et al., 1970), decreased plasma lactate levels could reflect increased neuronal activity, lactate consumption, and Aβ production in the brain, which also makes plasma lactate levels a potential serum biomarker for AD, T2D, or both. In humans, a small cohort study established that serum lactate levels decreased in symptomatic AD, yet the authors attributed this finding to alterations in muscle metabolism, not brain (Verri et al., 2018). Because the majority of the changes in lactate were found in the plasma and not the CSF, an alternative hypothesis is that T2D suppresses neuronal activity leading to the decreased CSF Aβ_40_ and Aβ_42_ concentrations. However, this is unlikely given that CSF Aβ was significantly decreased in T2D monkeys relative to healthy controls in spite of the unaltered CSF lactate concentrations. Thus, additional studies are needed in order to elucidate the role of plasma lactate in T2D and AD.

In the current study, we demonstrated that T2D monkeys have lower CSF acylcarnitine and amino acid concentrations (Figures 2, 3). Several studies demonstrated that circulating levels of amino acids are positively correlated with obesity, insulin resistance, metabolic dysfunction, and T2D in humans and rodents (Newgard et al., 2009; Wang et al., 2011; Solon-Biet et al., 2019). Although our data demonstrates a trend toward an increase in the BCAAs valine and isoleucine/leucine, no differences in plasma amino acid concentrations were detected in PreD or T2D monkeys. This may be explained by the fact that the T2D monkeys in this study were fed a well-controlled, balanced diet that did not replicate the traditional nutritional overconsumption seen humans with metabolic syndrome and T2D. Although the T2D monkeys were hyperglycemic, their insulin levels were unchanged, suggesting that the hyperglycemia may arise via a mechanism independent of insulin resistance. Studies reporting elevated plasma BCAAs in humans have involved obese and insulin resistant subjects (Newgard et al., 2009; Solon-Biet et al., 2019). Therefore, future studies should explore the relationship between plasma and CSF amino acid levels in a non-human primate model of dietary induced metabolic syndrome and T2D.

Another explanation for the difference in CSF AA levels could be that the T2D brain is overconsuming amino acids as fuel or rapidly increasing protein synthesis. While glucose is the primary source of energy for the brain, the brain can readily use fatty-acids as energy substrates; however, this typically occurs with decreased glucose availability, such as fasting or starvation (Costa et al., 1999). This could lead the brain to a state of hypermetabolic overconsumption if both glucose and fatty acid metabolism were upregulated. Furthermore, because many of the amino acids consumed by the brain are necessary for neurotransmitter biosynthesis or neurotransmission itself (Sperringer et al., 2017), we propose that increased amino acid consumption increases both synaptic activity and metabolic activity, leading to elevated Aβ production, oxidative stress, and Aβ aggregation. Our current data cannot discern if the decrease in amino acids and acylcarnitines in the CSF is due to increased oxidation, or by another means, such as altered amino acid transport (Supplementary Figure S3 and Supplementary Table S5), therefore additional studies will need to further elucidate mechanisms underlying the changes in CSF metabolites.

## Conclusion

The data presented here show that in the progression from healthy to PreD to T2D, the brain moves into a state of altered metabolism that results in an increase in glucose and lowering of amino acids and acylcarnitines in the CNS. Increased cerebral metabolism drives Aβ production and accelerates Aβ aggregation, which reciprocally escalates the disease cascades in T2D and AD. These findings shed further light on the metabolic link between T2D and amyloid pathology and how T2D progression could lead to AD-related pathology and cognitive decline.

## Data Availability

All datasets generated for this study are included in the manuscript and/or Supplementary Files.

## Ethics Statement

Animal subjects: The animal study was reviewed and approved by Wake Forest University Institutional Animal Care and Use Committee.

## Author Contributions

KK, DM, SM, CN, and OI conceived on the experimental design. KK, MP, WM, CN, OI, and SM performed the experiments. SD, KK, CN, OI, DM, and SM analyzed the data and wrote the manuscript.

## Conflict of Interest Statement

The authors declare that the research was conducted in the absence of any commercial or financial relationships that could be construed as a potential conflict of interest.

## Supplementary Material

FIGURE S1 T2D monkeys had significantly decreased levels of several essential, branched chain, and aromatic amino acids in the CSF. **(A–E)** T2D and PreD monkeys had significantly decreased glycine, proline, methionine, aspartate/asparagine, and tyrosine. **(F–J)** T2D monkeys had significantly decreased valine, phenylalanine, ornithine, citrulline, and arginine. **(K–O)** There were no differences in alanine, serine, leucine/isoleucine, histidine, or glutamine/glutamic acid between groups.

FIGURE S2 There were no differences in aminoacid concentrations between groups in the plasma. **(A–O)**Plasma amino acid concentrations were similar between Ctrl, PreD, and T2D monkeys.

FIGURE S3 Amino acids carried by large neutral amino acid or L-type transporters, but not A, N, or EAAT transporters, were decreased in the CSF of T2D monkeys. **(A,B)** Amino acids (AAs) carried by large neutral amino acid (LNAA)- or L-type-transporter were decreased in the CSF of T2D monkeys. **(C,D)** There were no differences in A-, N-, and EAAT-transporter AA concentrations in the CSF of PreD and T2D monkeys. **(F–J)** There were no differences in LNAA-, L-type-, A-, N-, and EAAT-transported amino acid concentrations in plasma of PreD or T2D monkeys compared to controls.

TABLE S1 Descriptive statistics of amino acid data (see Figure 2).

TABLE S2 Descriptive statistics of acylcarnitine data (see Figure 3).

TABLE S3 Descriptive statistics of CSF amino acid data (see Supplementary Figure S1).

TABLE S4 Descriptive statistics of plasma amino acid data (see Supplementary Figure S2).

TABLE S5 Descriptive statistics of amino acid data, grouped by AA transporter (see Supplementary Figure S3).

TABLE S6 Descriptive statistics of normalized CSF glucose, plasma glucose, CSF Aβ42, and plasma lactate with various CSF analytes. See Figure 4.
