# Supplementary figures and images for: Type-2-Diabetes Alters CSF but Not Plasma Metabolomic and AD Risk Profiles in Vervet Monkeys
Source: Front Neurosci. 2019 Aug 28;13:843. doi: 10.3389/fnins.2019.00843 (PMC6722201; doi:10.3389/fnins.2019.00843)

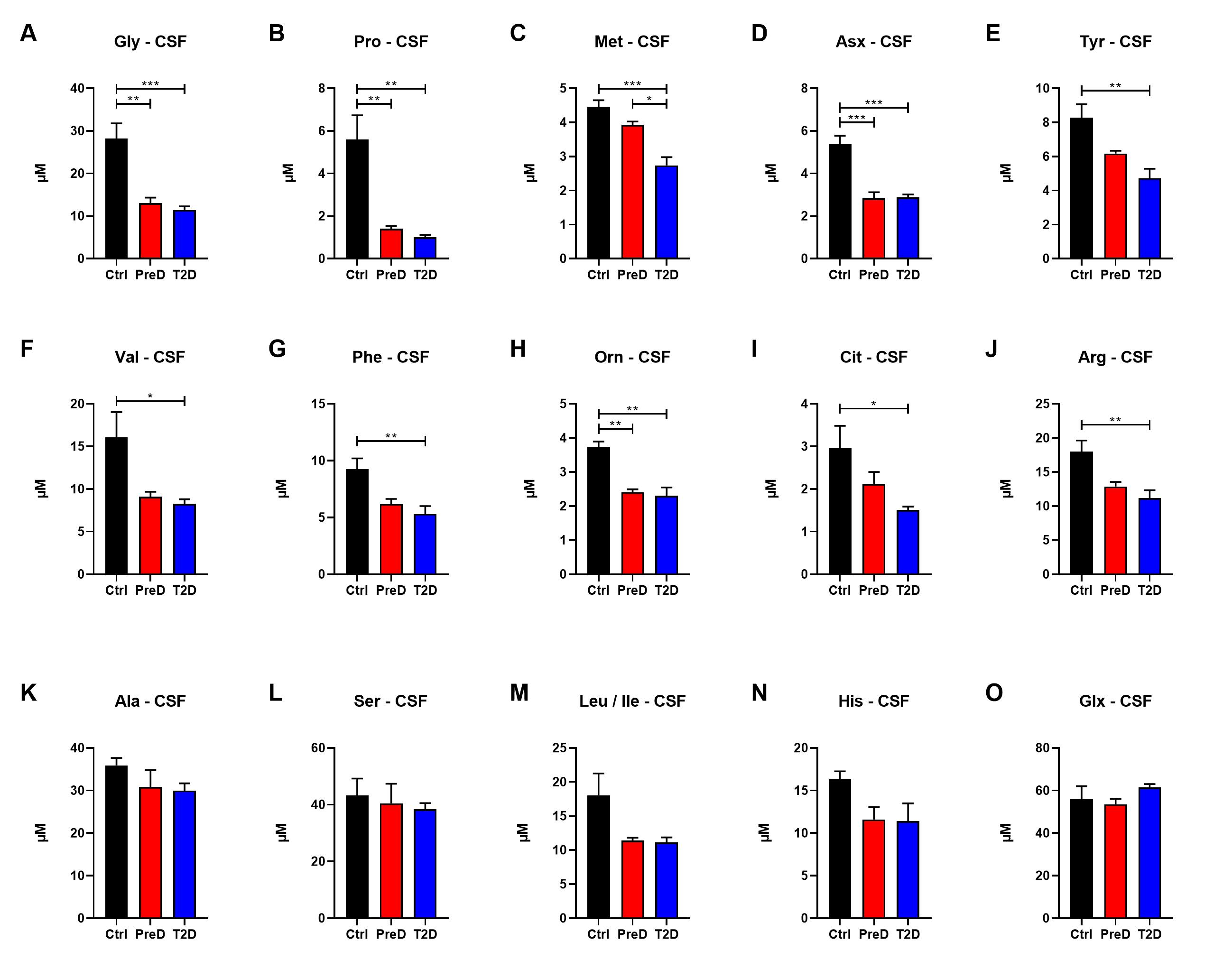

Supplement: FIGURE S1 — T2D monkeys had significantly decreased levels of several essential, branched chain, and aromatic amino acids in the CSF. (A–E) T2D and PreD monkeys had significantly decreased glycine, proline, methionine, aspartate/asparagine, and tyrosine. (F–J) T2D monkeys had significantly decreased valine, phenylalanine, ornithine, citrulline, and arginine. (K–O) There were no differences in alanine, serine, leucine/isoleucine, histidine, or glutamine/glutamic acid between groups. [file Data_Sheet_1.zip › Supplemental Figure 1.jpg]

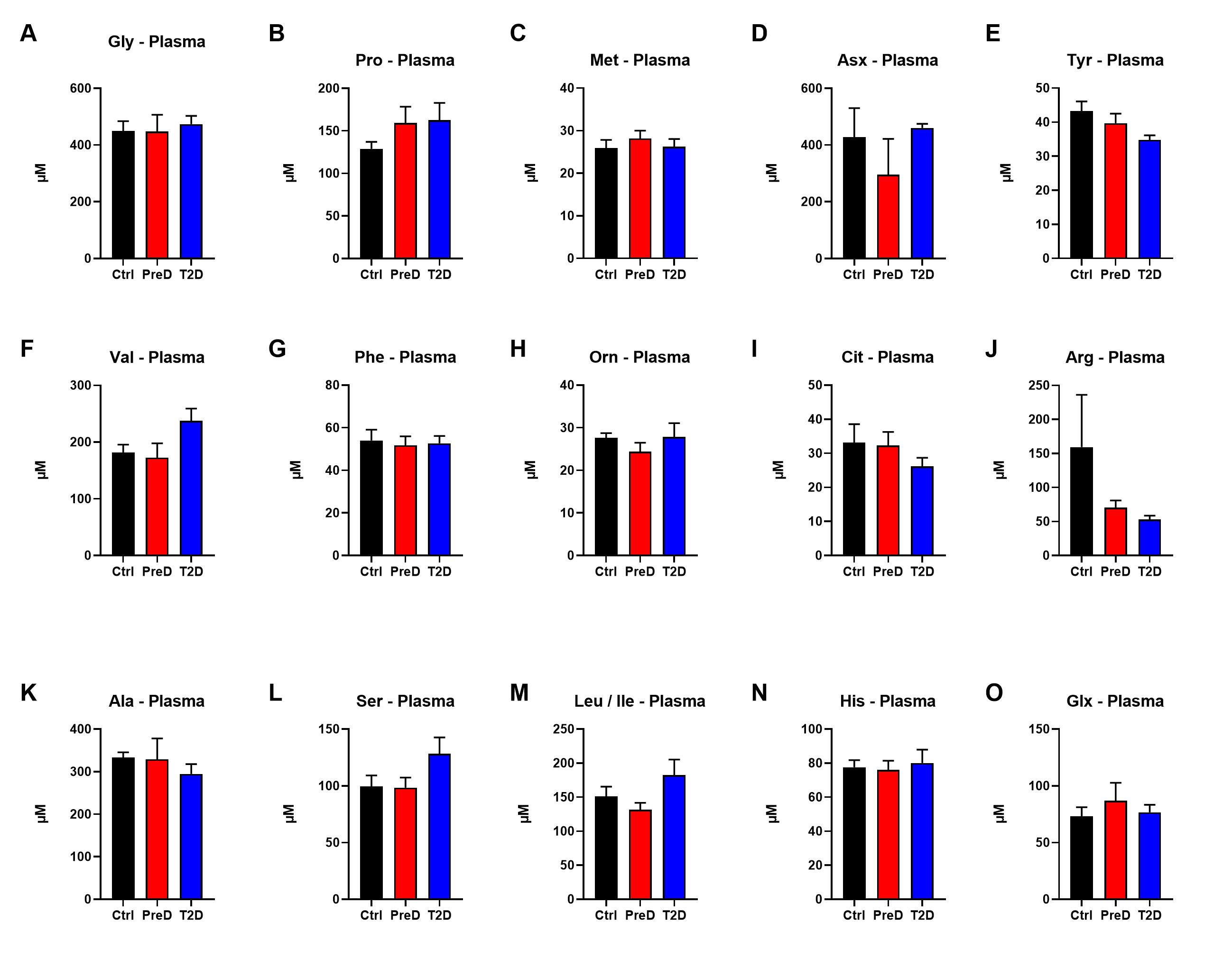

Supplement: FIGURE S1 — T2D monkeys had significantly decreased levels of several essential, branched chain, and aromatic amino acids in the CSF. (A–E) T2D and PreD monkeys had significantly decreased glycine, proline, methionine, aspartate/asparagine, and tyrosine. (F–J) T2D monkeys had significantly decreased valine, phenylalanine, ornithine, citrulline, and arginine. (K–O) There were no differences in alanine, serine, leucine/isoleucine, histidine, or glutamine/glutamic acid between groups. [file Data_Sheet_1.zip › Supplemental Figure 2.jpg]

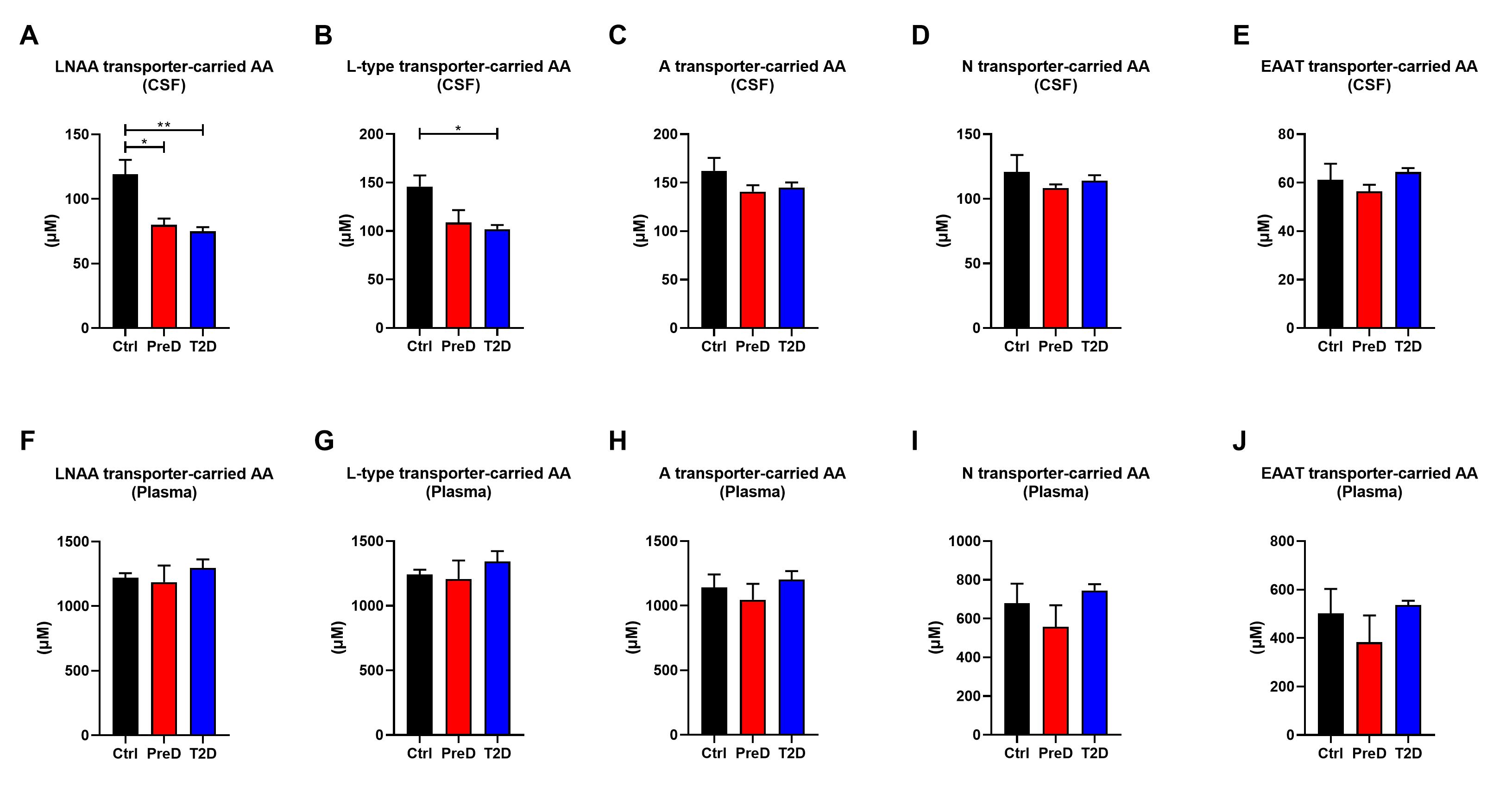

Supplement: FIGURE S1 — T2D monkeys had significantly decreased levels of several essential, branched chain, and aromatic amino acids in the CSF. (A–E) T2D and PreD monkeys had significantly decreased glycine, proline, methionine, aspartate/asparagine, and tyrosine. (F–J) T2D monkeys had significantly decreased valine, phenylalanine, ornithine, citrulline, and arginine. (K–O) There were no differences in alanine, serine, leucine/isoleucine, histidine, or glutamine/glutamic acid between groups. [file Data_Sheet_1.zip › Supplemental Figure 3.jpg]

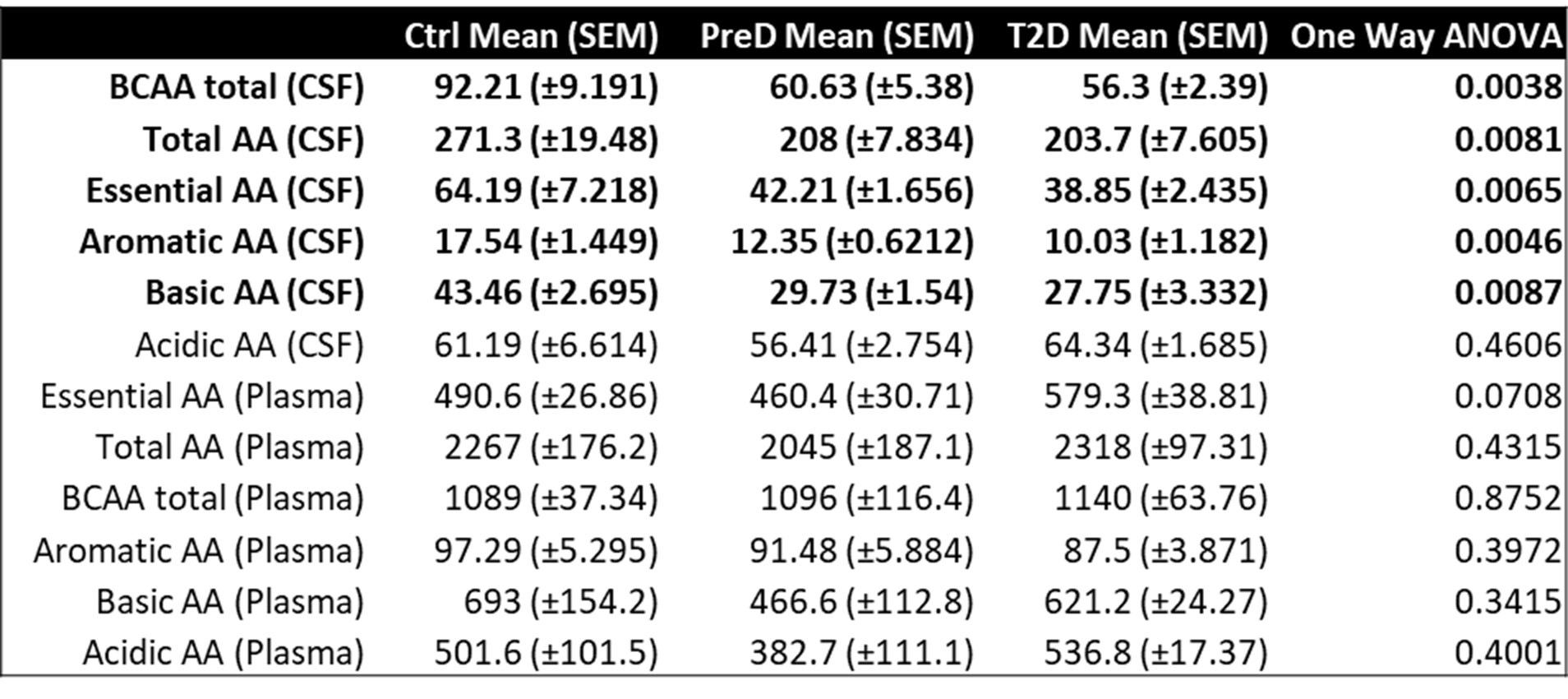

Supplement: FIGURE S1 — T2D monkeys had significantly decreased levels of several essential, branched chain, and aromatic amino acids in the CSF. (A–E) T2D and PreD monkeys had significantly decreased glycine, proline, methionine, aspartate/asparagine, and tyrosine. (F–J) T2D monkeys had significantly decreased valine, phenylalanine, ornithine, citrulline, and arginine. (K–O) There were no differences in alanine, serine, leucine/isoleucine, histidine, or glutamine/glutamic acid between groups. [file Data_Sheet_1.zip › Supplemental Table 1.jpg]

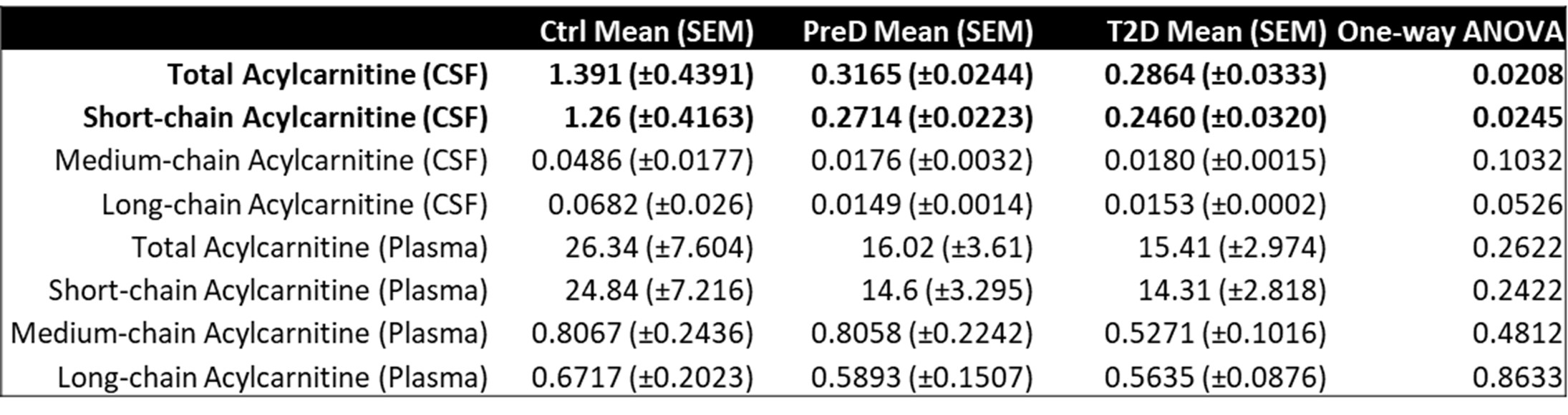

Supplement: FIGURE S1 — T2D monkeys had significantly decreased levels of several essential, branched chain, and aromatic amino acids in the CSF. (A–E) T2D and PreD monkeys had significantly decreased glycine, proline, methionine, aspartate/asparagine, and tyrosine. (F–J) T2D monkeys had significantly decreased valine, phenylalanine, ornithine, citrulline, and arginine. (K–O) There were no differences in alanine, serine, leucine/isoleucine, histidine, or glutamine/glutamic acid between groups. [file Data_Sheet_1.zip › Supplemental Table 2.jpg]

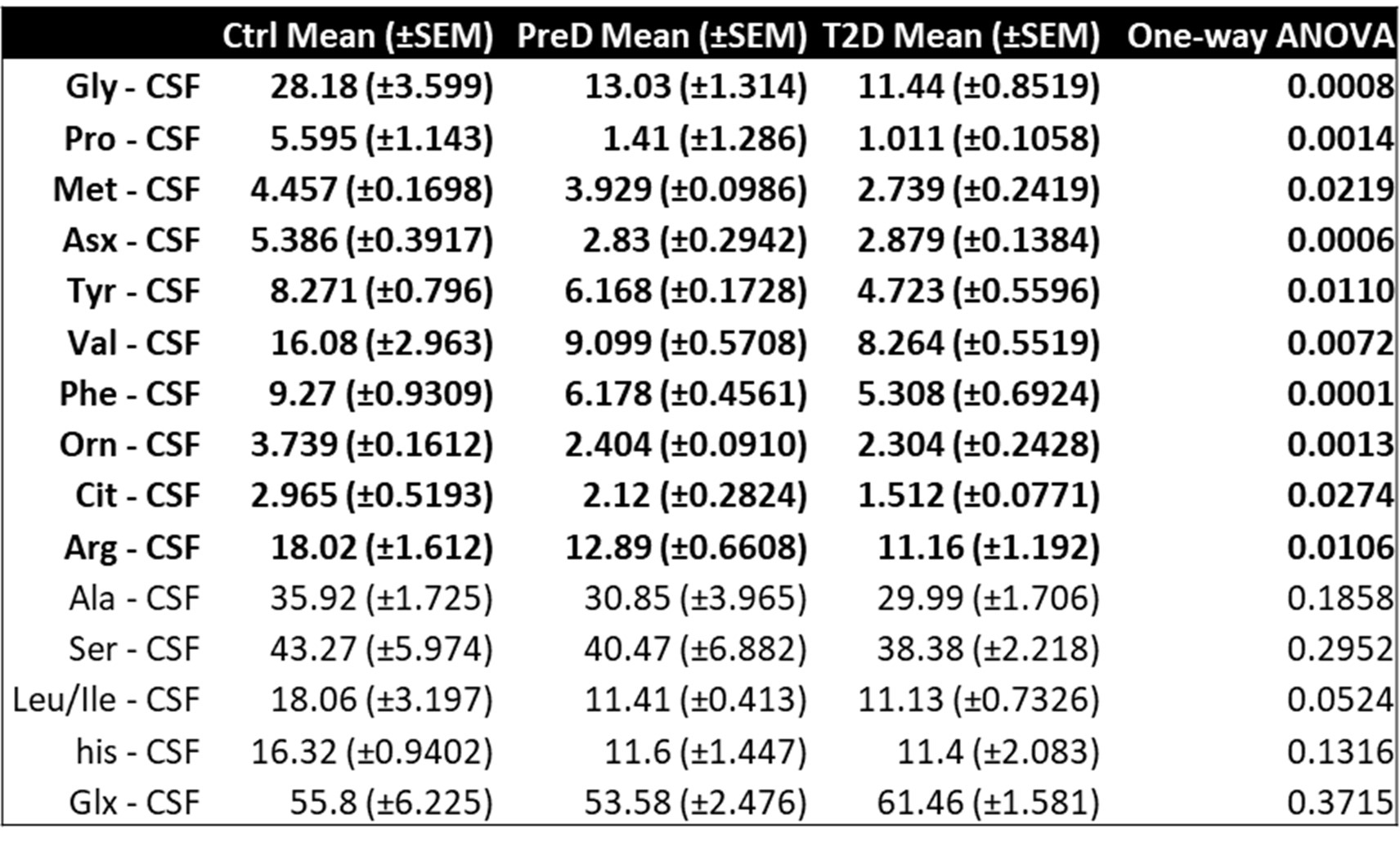

Supplement: FIGURE S1 — T2D monkeys had significantly decreased levels of several essential, branched chain, and aromatic amino acids in the CSF. (A–E) T2D and PreD monkeys had significantly decreased glycine, proline, methionine, aspartate/asparagine, and tyrosine. (F–J) T2D monkeys had significantly decreased valine, phenylalanine, ornithine, citrulline, and arginine. (K–O) There were no differences in alanine, serine, leucine/isoleucine, histidine, or glutamine/glutamic acid between groups. [file Data_Sheet_1.zip › Supplemental Table 3.jpg]

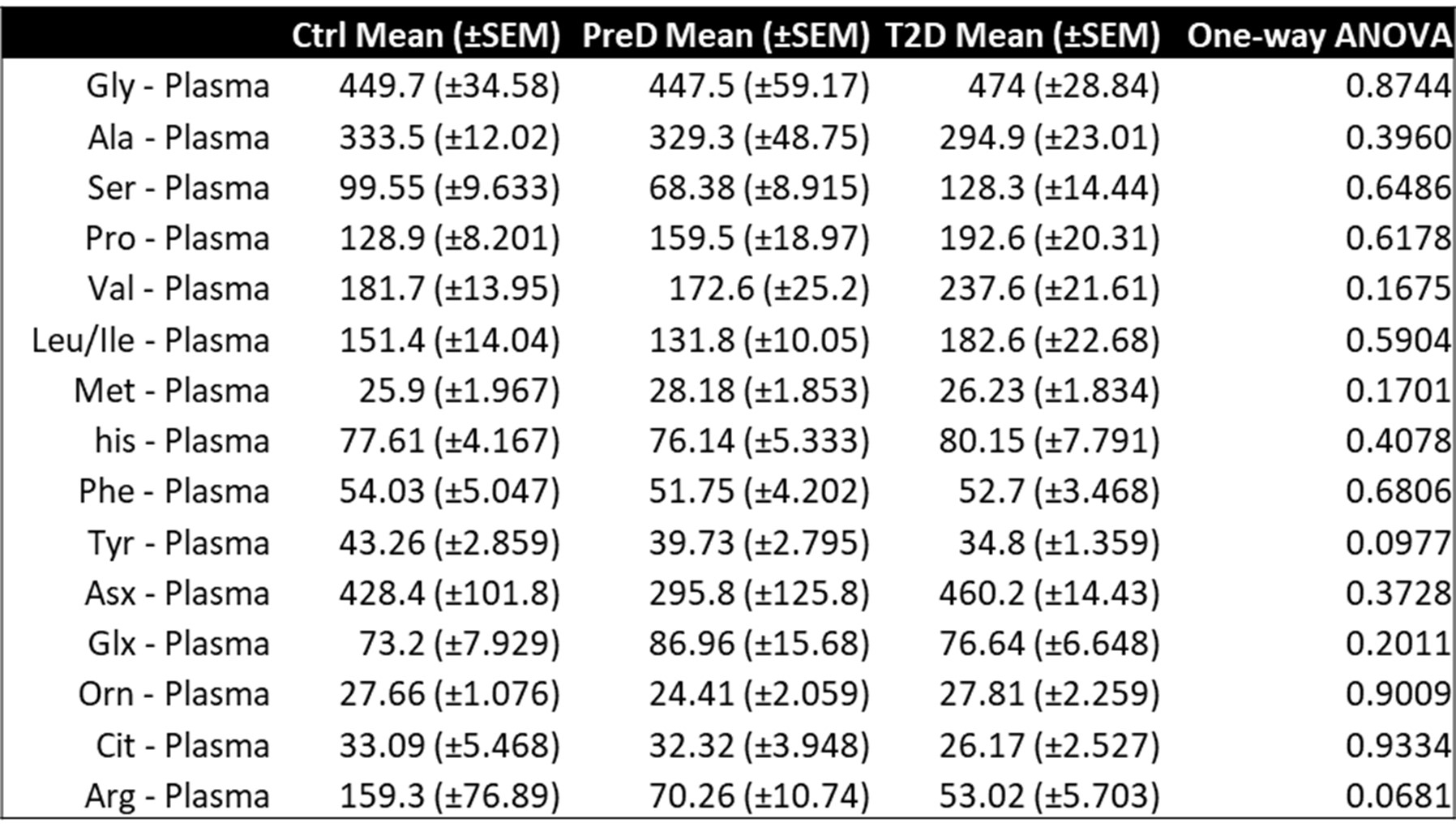

Supplement: FIGURE S1 — T2D monkeys had significantly decreased levels of several essential, branched chain, and aromatic amino acids in the CSF. (A–E) T2D and PreD monkeys had significantly decreased glycine, proline, methionine, aspartate/asparagine, and tyrosine. (F–J) T2D monkeys had significantly decreased valine, phenylalanine, ornithine, citrulline, and arginine. (K–O) There were no differences in alanine, serine, leucine/isoleucine, histidine, or glutamine/glutamic acid between groups. [file Data_Sheet_1.zip › Supplemental Table 4.jpg]

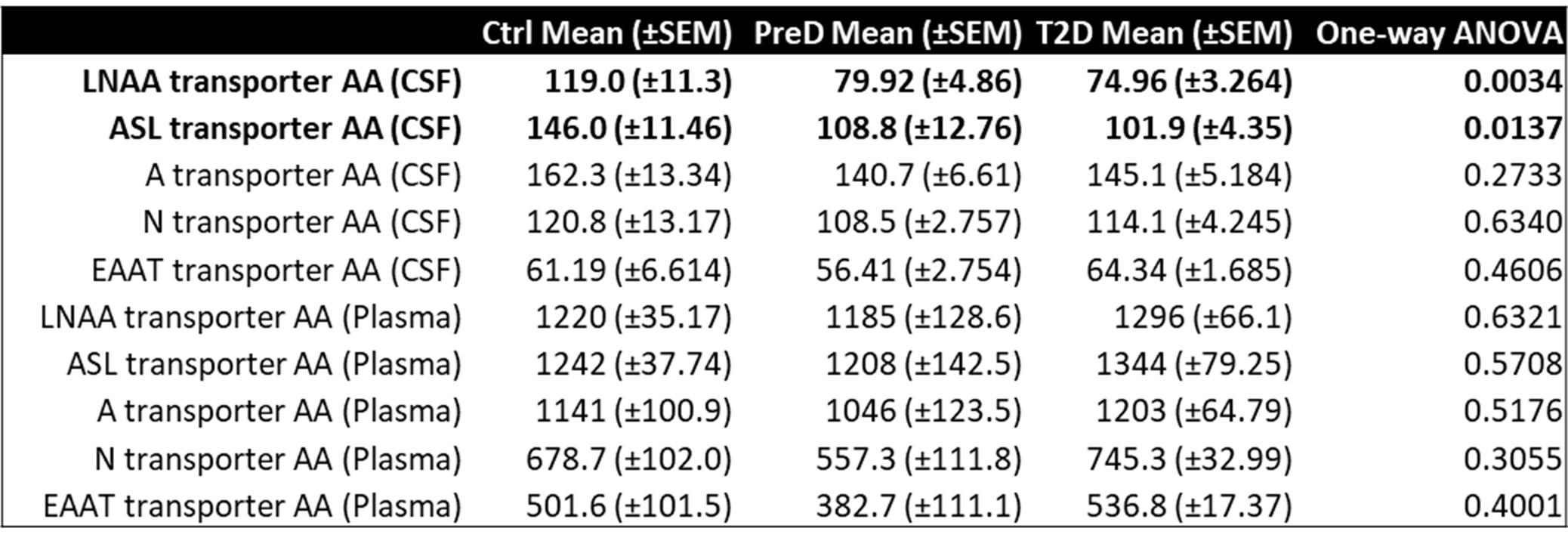

Supplement: FIGURE S1 — T2D monkeys had significantly decreased levels of several essential, branched chain, and aromatic amino acids in the CSF. (A–E) T2D and PreD monkeys had significantly decreased glycine, proline, methionine, aspartate/asparagine, and tyrosine. (F–J) T2D monkeys had significantly decreased valine, phenylalanine, ornithine, citrulline, and arginine. (K–O) There were no differences in alanine, serine, leucine/isoleucine, histidine, or glutamine/glutamic acid between groups. [file Data_Sheet_1.zip › Supplemental Table 5.jpg]

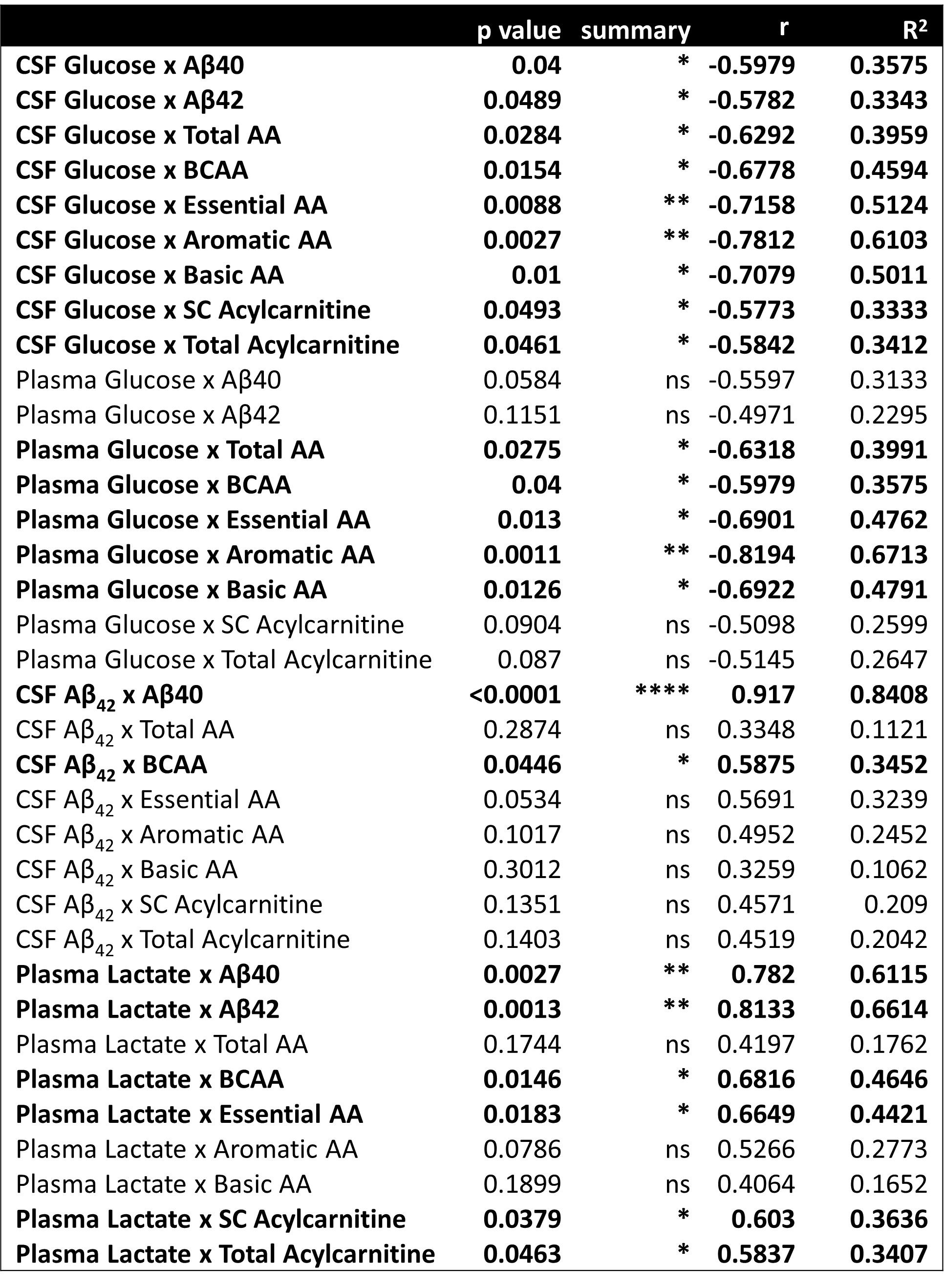

Supplement: FIGURE S1 — T2D monkeys had significantly decreased levels of several essential, branched chain, and aromatic amino acids in the CSF. (A–E) T2D and PreD monkeys had significantly decreased glycine, proline, methionine, aspartate/asparagine, and tyrosine. (F–J) T2D monkeys had significantly decreased valine, phenylalanine, ornithine, citrulline, and arginine. (K–O) There were no differences in alanine, serine, leucine/isoleucine, histidine, or glutamine/glutamic acid between groups. [file Data_Sheet_1.zip › Supplemental Table 6.jpg]
